# After-Results of Magnet Extraction

**Published:** 1910-11-26

**Authors:** 


### Ophthalmology.

### AFTER-RESULTS OF MAGNET EXTRACTION.

The value of the giant magnet devised by Haab for the removal from the interior of the eye of metallic foreign bodies is fairly well established; yet the after-results are only too often disappointing when compared with the immediate technical success. Dr. C. S. Bull* analyses eighteen cases in which he has been able to follow up the subsequent career of patients thus treated for foreign body in the vitreous or the fundus. It is, however, to be noted that most of the patients could not be traced, and it is quite possible that those who thus disappeared were those to whom complete and permanent relief had been afforded. It is instructive to read that in not one of the eighteen cases was permanent useful vision secured, and that enucleation was eventually necessary in ten of them. The author formulates several rules for the management of these important cases. The foreign body should be located, and an attempt at extraction made as soon as possible after the accident, in order to lessen the dangers of infection. When the wound of entrance is in the anterior segment of the eye, and the foreign body has been located in the vitreous or the fundus no attempt should be made to draw it forward by the Haab magnet through the wound of entrance. It is far wiser to make an incision through the sclera at the nearest point to the foreign body and extract it by means of the small magnet. Further, it is to be remembered that the shock to the eye of the original accident has to be taken account of; this is practically always severe, and it is important to appreciate how severe is the additional shock of applying the giant magnet and of drawing the foreign body forcibly through delicate tissues already seriously injured.

Prognosis is always unfavourable, from the great danger of sympathetic uveitis; and an eye containing a foreign body in its interior must always be regarded as in a dangerous condition. Certainly if infection has already begun, prompt enucleation is the only treatment that is admissible. Prognosis is slightly less grave when the foreign body is lodged in the vitreous than when it is in the retina or the ciliary body.

The giant magnet is a dangerous weapon in the hands even of the most expert, and great care must always be exercised in its application; for more injury may be done by the passage of the fragment of iron or steel through the tissues under the traction of the magnet than is caused by the original accident. If attempted extraction fail, it is best to enucleate the eye at once. Even after successful extraction useful vision will be permanently lost in the majority of cases, and enucleation will be necessary in order to save the fellow eye. The danger of sympathetic ophthalmia is ever present, and a general insidious uveitis may develop months, and even years, afterwards, and extend to the other eye, with disastrous consequences.

* *Medical Record*, August 27, 1910, p. 347.

